# Pathogenesis of Pulmonary Manifestations in ANCA-Associated Vasculitis and Goodpasture Syndrome

**DOI:** 10.3390/ijms25105278

**Published:** 2024-05-12

**Authors:** Evangelia Fouka, Fotios Drakopanagiotakis, Paschalis Steiropoulos

**Affiliations:** 1 Department of Respiratory Medicine, General Hospital G. Papanikolaou, Medical School, Aristotle University of Thessaloniki, 57010 Thessaloniki, Greece; evafouka@gmail.com; 2 Department of Respiratory Medicine, Medical School, Democritus University of Thrace, 68100 Alexandroupolis, Greece; steiropoulos@yahoo.com

**Keywords:** pathogenesis, ANCA-associated vasculitis, lung

## Abstract

Pulmonary manifestations of vasculitis are associated with significant morbidity and mortality in affected individuals. They result from a complex interplay between immune dysregulation, which leads to vascular inflammation and tissue damage. This review explored the underlying pathogenesis of pulmonary involvement in vasculitis, encompassing various forms such as granulomatosis with polyangiitis (GPA), microscopic polyangiitis (MPA), eosinophilic granulomatosis with polyangiitis (EGPA), and anti-GBM disease. Mechanisms involving ANCA and anti-GBM autoantibodies, neutrophil activation, and neutrophil extracellular trap (NETs) formation are discussed, along with the role of the complement system in inducing pulmonary injury. Furthermore, the impact of genetic predisposition and environmental factors on disease susceptibility and severity was considered, and the current treatment options were presented. Understanding the mechanisms involved in the pathogenesis of pulmonary vasculitis is crucial for developing targeted therapies and improving clinical outcomes in affected individuals.

## 1. Introduction

Vasculitis is a heterogeneous group of disorders with common pathophysiological mechanisms, characterized by inflammation of the blood vessels, which can present with a range of pathological features and clinical manifestations depending on the vessels affected [1]. The classification of vasculitis is based on the size of the affected blood vessels, with small-vessel vasculitis being the most prevalent form affecting the lungs and often associated with anti-neutrophil cytoplasmic antibodies [2]. Large- and medium-vessel vasculitis are uncommon and may involve the lungs; however, their clinical manifestations are poorly described [3]. Vasculitis may present as a primary disorder or secondary to another underlying disease [4].

Anti-neutrophil cytoplasmic antibody (ANCA)-associated vasculitis (AAV) is categorized into three types: granulomatosis with polyangiitis (GPA), microscopic polyangiitis (MPA), and eosinophilic GPA (EGPA, previously known as Churg–Strauss syndrome) [5]. Loss of tolerance to neutrophil primary granule proteins is characteristic of these disorders. At the same time, the occurrence of autoimmune mechanisms is typically confirmed by the detection of serum ANCAs against PR3 (PR3-ANCA) or MPO (MPO-ANCA) antigens [5]. These disorders primarily affect small blood vessels, including capillaries, arterioles, and venules [6]. Historically, the disease was considered rare. However, recent studies have indicated a prevalence rate of 300–421 per million people, probably attributed to survival rate increases and diagnostic confirmation [6].

Goodpasture syndrome, or anti-glomerular basement membrane (anti-GBM) disease, manifests as an autoimmune condition in which antibodies target epitopes of an autoantigen found in collagen IV, a key component of kidney and lung basement membranes [7].

This article offers an updated overview of the pathogenetic mechanisms involved in the pulmonary manifestations of primary small-cell vasculitis, namely AAV and anti-GBM disease.

## 2. Pulmonary Manifestations of Small-Vessel Vasculitis

The diagnosis of vasculitis can be difficult, as symptoms often overlap with those of other, more common conditions. This can be further complicated because the same vasculitis can affect different organs in different individuals, with the severity of organ involvement varying significantly from person to person [8]. The clinical presentation may appear rapidly in some cases, whereas in others, it may develop gradually over several weeks or months [9].

Numerous potential pulmonary manifestations of large-, medium-, and small-vessel vasculitis have been reported. Certain manifestations, such as pulmonary artery aneurysms and diffuse alveolar hemorrhage, are frequently associated with vasculitis pathology, making it easier to suspect this condition when it is discovered in imaging [10,11]. Vasculitis should also be suspected in patients with pulmonary hypertension and systemic complaints [12] or in those with evidence of subclavian, carotid, or renal artery stenosis [13]. Similarly, multiple pulmonary infarctions in patients without known risk factors for thromboembolic disease should raise suspicion of vasculitis [14]. Finally, constitutional symptoms, such as fever, weight loss, fatigue, and night sweats, or evidence of end-organ damage due to vascular inflammation and ischemia, such as stroke, vision loss, uveitis, or sensorineural hearing loss, should prompt consideration for the diagnosis of vasculitis [15].

### 2.1. GPA and MPA

Patients with AAV usually experience severe and occasionally life-threatening disease, although milder cases have also been reported [16]. Evidence of AAV can be found in any organ or tissue. However, GPA and MPA most commonly affect the upper and lower respiratory tracts and kidneys [17]. Common features of GPA include sinonasal disease, lower respiratory tract involvement with granulomatous inflammation or pulmonary hemorrhage, and glomerulonephritis [18]. Patients with MPA may present with some GPA symptoms, although without granulomatous inflammation and usually with more severe renal disease [19,20].

**Figure 1:**
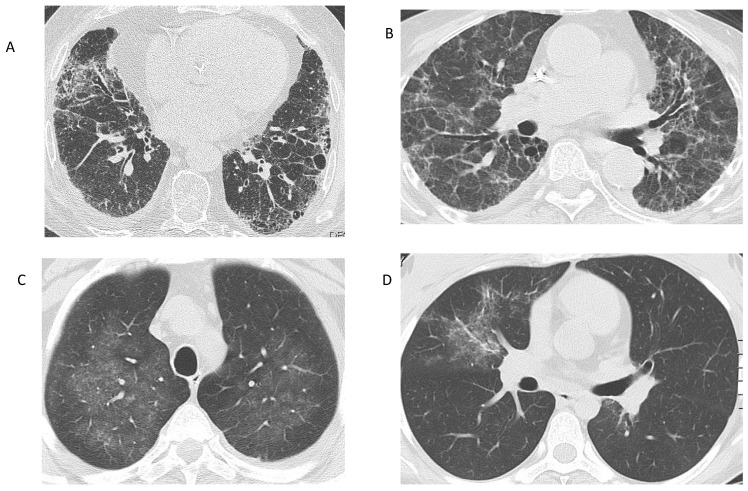
Radiographic findings in AAV. (**A**) Interstitial lung disease in a patient with MPA. Reticular opacities at the basal and peripheral lung segments, corresponding to a UIP pattern. (**B**) Interstitial lung disease in a patient with MPA, who presented with acute renal failure. Reticular changes and ground-glass opacities, suggestive of an NSIP pattern. (**C**) Ground-glass opacities in a patient with GPA: Bronchoalveolar lavage revealed alveolar hemorrhage. (**D**) Ground-glass opacity in the right upper lobe of a patient with EGPA, representing eosinophilic infiltration of the lung. (Images from the personal archive of Prof. Steiropoulos).

The pulmonary manifestations of these types of AAV may vary widely, ranging from being asymptomatic to experiencing cough, shortness of breath, chest pain from pleural irritation or hemoptysis, and severe, life-threatening respiratory failure [21]. The key histopathological feature of GPA is granulomatous inflammation, which may be identified in the lungs in up to 50% of patients as singular or multiple nodules that tend to cavitate due to central necrosis. Therefore, chest radiography or computed tomography may be crucial for detecting lung involvement, particularly when respiratory symptoms are mild or absent (Figure 1) [22]. Transbronchial or surgical biopsies may be warranted to rule out malignant or infectious conditions that may mimic vasculitis or to confirm the diagnosis in cases of recognition of isolated lung nodules without evidence of involvement of other organs [23]. Histopathology of these lesions typically reveals fibrinoid necrosis, neutrophilic microabscesses, palisading histiocytes, and giant cell accumulation that form a granulomatous inflammation pattern known as ‘geographic necrosis’ [24]. In addition, pathological findings may include focal vasculitis, thrombosis, and vascular lumina obliteration [25]. Cavitary lung lesions are prone to colonization by fungi such as *Aspergillus fumigatus*, posing a diagnostic problem as the radiographic features of fungal infections are often similar to those of GPA [26].

Involvement of the tracheobronchial area is particularly common in GPA and can affect any part of the airway, with subglottic lesions being the most common finding [27]. These lesions are typically segmental and focal and are characterized by inflammatory erosions of the mucosa, which may lead to cartilage involvement, tracheomalacia, or stenosis [28]. Tracheobronchial involvement in GPA may manifest with various symptoms, including cough, dyspnea, stridor, localized wheezing, and hemoptysis [29].

Diffuse alveolar hemorrhage (DAH) is a severe lung condition associated with AAV, with mortality rates ranging from 10–25%, representing a poor prognostic factor [30]. DAH affects 12–29% of patients with MPA [31], compared to GPA and EGPA, which involve 22-30% and 4% of patients, respectively [32]. DAH in AAV indicates pulmonary capillaritis, with predominantly neutrophilic inflammation and fibrinoid necrosis of the capillary walls, which may lead to erythrocyte extravasation, subsequent gas exchange impairment, and acute presentation with nonspecific symptoms, such as dyspnea, hypoxemia, or anemia [33]. Of note, radiographic findings may be normal in up to 50% of cases, in which computed tomography may be required to reveal signs of alveolar hemorrhage, such as bilateral, ground-glass opacities, crazy-paving patterns, or intra- and interlobular thickening [34]. Bronchoalveolar lavage (BAL) is the gold standard procedure for diagnosing DAH, but also for ruling out infections that may present with similar symptoms or radiographic findings, especially in immunocompromised patients [35,36]. It is also advisable to exclude other potential causes that can cause capillaritis, such as non-inflammatory vascular, cardiac, respiratory, drug-induced, neoplastic, and other autoimmune diseases, before confirming the diagnosis of AAV-related DAH [37].

In the past two decades, there has been an increasing number of cases of interstitial lung disease (ILD) reported, which can be either diagnosed at the same time as or even earlier than AAV manifestations, most commonly in the context of MPA [38]. Unlike DAH, ILD-related AAV can progress slowly and insidiously for years with nonspecific symptoms such as progressive dyspnea and cough [39]. Although velcro crackles are typically heard on physical examination, digital clubbing is rare [40]. Common findings on high-resolution computed tomography (HRCT) of ILD associated with MPA include ground-glass (23–94%) and reticular opacities (41–77%), interlobular septal thickening (41–71%), parenchymal consolidations (23–78%), and honeycombing, with the majority of patients (50–71% of cases) presenting the radiographic pattern of usual interstitial pneumonia (UIP) (23–52% of cases), followed by nonspecific interstitial pneumonia (NSIP) pattern (7–31%) and rarely desquamative interstitial pneumonia (up to 14%) [41]. Pulmonary function tests typically reveal a restrictive disorder and a decrease in diffusing capacity for carbon monoxide (DLCO) [42].

Patients initially diagnosed with idiopathic pulmonary fibrosis (IPF) may have positive ANCAs, particularly MPO-ANCAs, during the course of the disease, found in 8.5% of them at presentation, while after five years, 24.3% of them will eventually develop other features of MPA [43]. A retrospective study conducted in North America, including 745 patients with IPF, demonstrated that 25–33% of patients who were initially diagnosed with IPF and MPO-ANCA positivity developed vasculitis symptoms within a median follow-up period of 18 months [44]. Research indicates that approximately 10% of patients diagnosed with ANCA-negative IPF are expected to seroconvert during follow-up [45,46]. Furthermore, there have also been reports of patients with isolated pulmonary fibrosis who exhibit positive ANCA testing, without developing apparent systemic manifestations [47]. These findings align with the ACR/EULAR MPA classification criteria, where a positive test for P-ANCA or MPO-ANCA (+6 points) would suffice to fulfill the criteria for MPA (≥5 points) [48]. Patients with IPF and a UIP pattern on HRCT present a higher independent risk of developing clinically overt MPA [49]. Therefore, patients with UIP/IPF and positive MPO-ANCA test results should be carefully monitored and provided with immunosuppressive therapy when active MPA develops.

### 2.2. EGPA

EGPA is characterized by asthma, eosinophilia, and vasculitis in many cases. Although categorized as a form of AAV, it has fewer similarities with GPA and MPA concerning its genetic, pathogenetic, and clinical features and is typically considered a separate entity [20]. EGPA is primarily characterized as a type-2 related entity, with clinical manifestations of late-onset asthma in nearly all patients (95–100%) [50]. Asthma diagnosis usually precedes the diagnosis of EGPA for many years and approximately half of patients experience severe or uncontrolled asthma symptoms that may require high doses of glucocorticoids to maintain control of the disease [51]. In EGPA, asthma is frequently associated with chronic rhinosinusitis (80%), atopy (25%), blood eosinophilia (up to 95%), and ANCA positivity (10–40%), with antibodies often directed against the MPO antigen [52]. Two primary clinicopathological subsets of EGPA can be differentiated: one characterized by positive ANCA and predominant vascular symptoms (such as glomerulonephritis, purpura, and mononeuritis multiplex) and the other by negative ANCA and prominent eosinophilic symptoms (such as lung infiltrates and cardiomyopathy) [53]. Severe cases may show radiological abnormalities, such as peripheral ground-glass opacities, consolidation, bronchial thickening, or pleural effusions, while lung function tests reveal airway obstruction with a bronchodilator response and preserved or increased DLCO [54]. Histopathological findings of pulmonary nodules biopsies may show eosinophilic necrosis, while a high percentage of eosinophils in the BAL differential cell count indicates eosinophilic pneumonia as a manifestation of EGPA [55].

### 2.3. Goodpasture Syndrome

This condition is historically characterized by the presence of three distinct features: DAH, glomerulonephritis, and circulating anti-GBM antibodies, with concurrent lung and renal involvement in most cases [56]. Immunopathogenesis of the disease is caused by autoantibodies directed against a component of type IV collagen found in the capillary basement membranes of the lungs and kidneys, referred to as anti-GBM antibodies [57].

Pulmonary symptoms are typically present at the disease onset or shortly afterwards [58]. Hemoptysis, ranging from severe and life-threatening to subtle diffuse hemorrhage, is commonly observed with pulmonary involvement and is characterized by extensive bilateral airspace consolidation [59]. Hemoptysis is more common in younger patients with anti-GBM disease and concurrent severe renal and pulmonary symptoms at presentation. In contrast, patients over 50 years of age usually present with glomerulonephritis only and experience a less severe disease course [7].

## 3. Pathogenetic Mechanisms of Pulmonary Manifestations of AAV

### 3.1. Genetic and Epigenetic Factors

The relatively low prevalence of isolated AAV accounts for the lack of robust genetic associations in large-scale gene studies. However, the use of cohorts that included patients with GPA, MPA, and sometimes EGPA revealed AAV associations with some major histocompatibility complex (MHC) genes, particularly the HLA-DPB1*04:01 allele in PR3-AAV [60]. The first genome-wide association study (GWAS) on AAV conducted by the European Vasculitis Genetics Consortium [61] demonstrated that GPA and MPA exhibit genetic differences, with strong associations of both MHC and non-MHC gene associations, not with the clinical syndromes themselves, but with ANCA specificity. The Vasculitis Clinical Research Consortium [62] confirmed these associations and provided the first evidence for genetic variants, such as those in PTPN22, common to both PR3-AAV and MPO-AAV. Other reported variants, such as PRTN3 (encoding PR3 genes), are more specific, suggesting that altered circulating PR3 availability is a key driver of the loss of tolerance to PR3 and subsequent development of PR3-AAV [63]. Similarly, the results of a large GWAS on EGPA [64] revealed 11 loci that are linked to EGPA, identifying two genetically distinct subtypes, MPO-ANCA + EGPA and ANCA–EGPA, which correspond to the clinical differences observed in these disease groups [65]. Furthermore, some of the identified loci were associated with eosinophil counts, suggesting that an increased risk of eosinophilia contributes to EGPA susceptibility.

### 3.2. The Role of Infections and Microbiome

Although some observational studies have implicated infectious triggers in AAV pathogenesis [6], the precise infectious agents involved remain unclear. The use of in vitro and in vivo animal model studies suggested several pathogenetic pathways by which infection might promote the loss of tolerance in AAV. These mechanisms include autoantigen exposure via the formation of neutrophil extracellular traps (NETs) [66], molecular mimicry (i.e., microbial antigens sharing sequence similarity with a host protein) [67], and priming of neutrophils for ANCA-induced activation [68]. ANCA antibodies have been reported to be positive in patients with subacute bacterial endocarditis and combined with positivity for antinuclear or antiphospholipid antibodies [69].

Nasal microbiome studies have revealed dysbiosis as a common trait in active AAV, which tends to normalize upon immunosuppressive treatment and in quiescent disease. However, the reported microbiome profiles differ considerably among patients [70]. Many studies have focused on *S. aureus*, with reports of increased rates of nasal carriage in patients with GPA and frequent relapses [71]. Experimental data implicate a plasmid-encoded 6-phosphogluconate dehydrogenase sequence from some *S. aureus* strains that induces molecular mimicry in MPO-AAV [72]. The focus on *S. aureus* results from the observation that approximately two-thirds of patients with GPA are chronic nasal carriers of this microbe [71]. *S. aureus* carriage is associated with an increased risk of AAV pulmonary exacerbations, and trimethoprim-sulfamethoxazole treatment effectively reduces the frequency of non-severe relapses [73,74].

Alterations in the gut microbiota have also been observed in patients with GPA [75]. A reduction in short-chain fatty acid (SCFA)-producing bacterial populations has been associated with kidney injury in AAV [76]. As many AAV patients report symptoms of infection in the weeks before disease onset, the findings mentioned above suggest a potential role for molecular mimicry in the development of autoimmune disease and AAV.

### 3.3. Common Pathogenetic Mechanisms in AAV

The pathogenesis of AAV overlaps, to some extent, with that of MPA, GPA, EGPA, and drug-induced AAV. These two main types of ANCA exhibit distinct cellular localization patterns, which can be identified using indirect immunofluorescence. One type, known as perinuclear ANCA (p-ANCA), is characterized by staining around the nucleus, whereas the other type, known as cytoplasmic ANCA (c-ANCA), exhibits diffuse staining of the cytoplasm, with the primary antigens targeted by p-ANCA and c-ANCA being MPO and PR3, respectively [77]. In addition to MPO and PR3, ANCAs have the potential to target various other neutrophil-derived molecules, including α-enolase, azurocidin, bactericidal permeability-increasing protein (BPI), cathepsin G, elastase, defensin, lactoferrin, lysosome-associated membrane glycoprotein 2 (LAMP2), and moesin; however, these ‘minor’ ANCAs generally exhibit low pathogenicity and are not typically associated with vasculitis [78].

Although the precise mechanisms underlying the loss of tolerance in AAV are not fully understood, several pathways have been proposed. In patients with AAV, reduced degradation of NETs has been linked to the production of ANCAs [79]. Although NETs are essential components of the innate immune system that help defend against infections, the formation of abnormal NETs that are resistant to degradation may modify the antigenicity of MPO or PR3 proteins, resulting in the development of MPO-ANCA or PR3-ANCA antibodies [80]. Upon infection, the production of pro-inflammatory cytokines, such as tumor necrosis factor (TNF)α and interleukin (IL)-1β, prime neutrophils to express target antigens that bind with ANCAs. The concomitant binding of ANCAs to the Fcγ receptors of neutrophils results in the excessive activation of neutrophils, which is eventually responsible for vascular injury and ischemia, manifesting in the lung as alveolar capillaritis. This effect is induced via excessive cytokine production, release of reactive oxygen species (ROS), lytic enzymes, and eventually NETs formation, leading to a vicious cycle [81,82]. Moreover, infection may also trigger the differentiation of naive T cells into T helper 17 (Th17) cells and the release of IL-17, which is a strong inducer of tissue neutrophilia, via the upregulation of the production of pro-inflammatory cytokines such as TNFα and IL-1β from macrophages [83]. Impaired clearance of apoptotic neutrophils may also result in prolonged exposure of autoantigens to circulating antigen-presenting cells [84].

Activation of the complement system is of paramount importance in the pathogenesis of AAV. In a mouse model of ANCA-mediated glomerulonephritis, the binding of C5a to its receptor on the neutrophil cell surface has been demonstrated to prime neutrophils for ANCA-induced respiratory burst [85]. In addition, C5a induces the release of tissue factor from neutrophils, leading to hypercoagulability in patients with AAV [86]. Similarly, elevated levels of C3a and C5a in the serum have been observed in patients with active AAV, suggesting activation of the alternative complement pathway and neutrophil priming [87].

Dendritic cells are also implicated in presenting MPO-containing NETs to CD4+ T cells, which subsequently induce the differentiation of B cells into MPO-ANCA-producing plasma cells [88]. Moreover, patients with AAV tend to have lower levels of and/or dysfunctional B and T regulatory lymphocytes, which, under normal conditions, suppress the proliferation of autoreactive T cells [89,90].

### 3.4. Granuloma Formation in GPA

**Figure 2:**
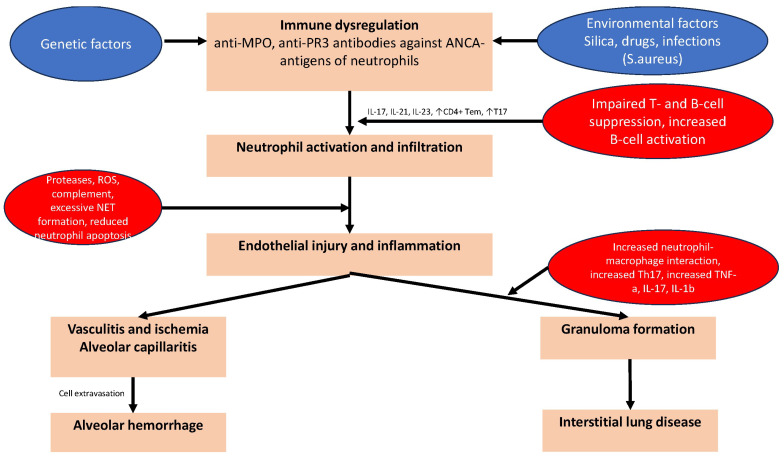
The pathogenetic mechanisms of pulmonary manifestations of GPA- and MPA-vasculitis. Common pathogenetic mechanisms in AAV are neutrophil activation and infiltration, which induce the formation of neutrophil extracellular traps (NETs), molecular mimicry, and priming of neutrophils for ANCA-induced activation. Genetic and epigenetic factors leading to immune dysregulation and the reduced degradation of NETs have also been linked to the production of ANCAs. Infection may trigger the differentiation of naive T cells into T helper 17 (Th17) cells and the release of IL-17, which induces tissue neutrophilia. Impaired clearance of apoptotic neutrophils may also result in prolonged exposure of autoantigens to circulating antigen-presenting cells leading to endothelial injury and inflammation. Vasculitis and subsequent ischemia, alveolar capillaritis/hemorrhage and granuloma formation are the primary pulmonary manifestations of GPA and MPA.

In the context of GPA, necrotizing granuloma formation is linked to infection, possibly by *S. aureus* [91]. This pathogen is thought to activate tissue-resident macrophages in the bronchial epithelium via toll-like receptors, leading to the release of pro-inflammatory cytokines, including TNF-α and IL-1β. Cytokine release induces the recruitment of neutrophils and monocytes from the blood into the developing lesions. Moreover, the recruited neutrophils also release ROS and lytic enzymes. These substances aid in the lysis of pathogens, ultimately leading to the formation of necrotic cores within the lesions. PR3 antigens on the surface of apoptotic neutrophils interfere with the macrophage phagocytosis of these cells [92]. An augmented Th17 response also contributes to granuloma formation surrounding the necrotic region, and increased IL-17 production has been demonstrated in both animal models and patients with active GPA [93,94]. The pathogenetic mechanisms of pulmonary manifestations of GPA- and MPA- vasculitis are shown in Figure 2.

### 3.5. EGPA Pathogenesis

EGPA is classified as a Th2-cytokine-mediated disease, and Th2 cytokines (such as IL-4, IL-5, and IL-13) and CCL26, a chemokine released from vascular endothelial cells, are responsible for eosinophil infiltration into tissues [95].

IL-5 plays a critical role in eosinophil extravasation. This cytokine is primarily synthesized by Th2 cells during allergen-triggered acquired immunity [96]. The differentiation of eosinophils, facilitated by IL-5, results in the production of IL-25, further fostering IL-5 production by Th2 cells [97]. Type 2 innate lymphoid cells (ILC2s) can serve as an alternative source of IL-5 in innate immunity [96]. ILC2s generate IL-5 in response to IL-33, which is released from endothelial cells and airway epithelium after infection. ILCs participate in mucosal immunity and tissue repair by releasing cytokines during the initial immune response [98] and are implicated in the pathogenesis of EGPA, as well as in allergic conditions [99].

In EGPA, eosinophils secrete eosinophilic granules, including eosinophilic neurotoxin, major basic proteins, and eosinophilic cationic proteins, which contribute to tissue damage [100]. Each of these cationic proteins is associated with different organ damage, such as vascular endothelial damage with eosinophilic peroxidase, cardiotoxicity and fibrosis with eosinophilic cationic protein, and neurotoxicity with eosinophilic neurotoxin. Cytolytic degranulation of eosinophils occurs through eosinophil apoptosis, but also through an active cytolysis mechanism, termed eosinophil extracellular trap cell death (EETosis) [101]. Eosinophil extracellular traps are composed of mitochondrial DNA scaffolds and a mixture of granule proteins. They contribute to the pathogenesis of eosinophilic diseases, including EGPA, by producing galectin-10, a cationic protein associated with the severity of EGPA vasculitis and IL-5 levels [96].

In EGPA, approximately 50% of patients test positive for MPO-ANCAs, and the presence of these autoantibodies is associated with kidney involvement, but inversely correlated with heart involvement [20]. ANCA positivity in EGPA correlates more with vasculitis manifestations, including alveolar hemorrhage, whereas ANCA negativity correlates with eosinophilic lung infiltrates [102]. Although eosinophil peroxidase shares 68% of its amino acid identity with neutrophil MPO, the mechanism by which MPO-ANCAs are produced in EGPA patients remains unclear [103]. Patients with MPO-ANCA positivity have been observed to exhibit higher frequencies of renal involvement, elevated serum C-reactive protein levels, and increased disease activity compared to those who tested negative for anti-lactoferrin antibodies [104]. Lactoferrin, an endogenous compound found in neutrophil-specific granules secreted upon their activation, has been shown to function as an inhibitor of NET formation [105]. In EGPA, targeting lactoferrin by anti-lactoferrin antibodies results in an increase in NET formation when neutrophils are activated [106]. The association between anti-lactoferrin antibodies and NET-related disease activity suggests that these antibodies play a significant role in the pathogenesis of this disorder.

### 3.6. The Role of Drugs in AAV

In past decades, drug-induced vasculitis (DIV) was poorly comprehended and defined empirically, with undefined terms, such as leukocytoclastic vasculitis, allergic vasculitis, hypersensitivity vasculitis, and serum sickness [107]. Traditionally, DIV has been characterized by ANCA positivity, with a specific drug identified as the suspected cause of the disease while excluding other types of vasculitis [108]. However, no clear definition has been proposed, and the 2012 International Chapel Hill Consensus Conference classified DIV as vasculitis associated with probable etiology [109]. So far, the drugs associated with DIV are primarily from all pharmacologic categories, mainly including anti-thyroid drugs and TNF-α inhibitors [110,111].

Although primary AAV and DIV share some common pathways in their pathogenesis [112], the exact mechanism underlying DIV remains poorly understood. Genome-wide association studies have identified several genes that contribute to DIV susceptibility, with MHC class II genes being the most strongly associated [113]. Genetic factors are more closely linked to ANCA specificity than to clinical manifestation, with PR3-ANCA associated with HLA-DP and MPO-ANCA associated with HLA-DQ [61]. Epigenetic modifications such as histone H3 lysine 27 trimethylation (H3K27me3) and DNA methylation have been found to play a role in AAV pathogenesis [114,115]. Some drugs, such as hydrazine, inhibit DNA methylation and induce self-reactivity in T cells, leading to the production of autoantibodies by activated T cells and plasma cells [116]. These studies suggest that abnormal epigenetic modifications are probably linked to the inappropriate expression of PR3 and MPO in patients with DIV. Infectious factors may stimulate neutrophils to form NETs, the persistence of which can damage tolerance to MPO and generate MPO-ANCA formation. As mentioned above, hydralazine can also significantly induce the formation of NETs [117]. However, some drugs, such as minocycline and clozapine, do not significantly induce NET formation or impair NET degradation [63]. This may suggest that some unknown mechanisms are also involved in the pathogenesis of DIV.

## 4. Pathogenetic Mechanisms of Goodpasture Syndrome

The pathogenesis of Goodpasture’s syndrome involves complex autoimmune mechanisms. GBM typically does not express epitopes that induce anti-GBM antibody production. However, under the influence of various factors, the hexameric structure of GBM, consisting of alpha 3, alpha 4, and alpha 5 chains, may become altered, exposing cryptic epitopes and triggering an autoimmune response [118]. Epitopes come in two categories: linear, with a sequential amino acid arrangement, and conformational, with a non-linear sequence. Both forms can be targeted by specific antibodies (B-cell epitopes). This leads to inflammation, the release of reactive oxygen species, activation of complement, modifications in GBM structure with gap formation, and subsequent cellular infiltration, resulting in crescent formation, glomerular dysfunction, and vascular necrosis [118]. When affecting the lungs, anti-GBM antibodies attack the hexameric structure of the alveolar basement membrane [56]. The resulting gap formation leads to extravasation of red blood cells into the alveoli, which can present as alveolar hemorrhage [56,119].

Cellular immunity, particularly involving T cells, plays a significant role in the pathogenesis of Goodpasture’s syndrome [120]. T cells can influence both B cell function and antibody production, and their dysregulation has been implicated in disease initiation and progression. T cells not only aid in B cell activation and maturation, but also identify antigenic determinants that stimulate Th1 and Th17 cell proliferation (T cell epitopes) [120,121]. T cell epitopes are presented by major histocompatibility complex molecules that trigger T cell immune responses [120,122,123].

Various factors such as infection, hydrocarbon exposure, smoking, or other environmental influences can induce conformational changes, exposing the epitopes to antibodies [124,125]. Specific HLA alleles (such as HLA-DRB1*1501 and HLA*DRB1*1502) may influence susceptibility to Goodpasture’s syndrome [126,127]. Additionally, certain genetic variants, such as HLA B7, have been associated with disease severity, while others, such as DRB1*0701 and DRB1*0101, have shown protective effects [128,129].

Patients with both anti-GBM and ANCA antibodies, often presenting as double-positive, exhibit a more severe clinical phenotype and worse outcomes compared to those with only anti-GBM antibodies or ANCA antibodies alone [130]. The ANCA antibodies are mainly anti-MPO. This association suggests potential shared pathogenetic mechanisms between anti-GBM disease and ANCA-associated vasculitis [130,131].

## 5. Treatment of Pulmonary Manifestations in AAV

The treatment of AAV typically involves a combination of immunosuppressive medications to control inflammation, prevent organ damage, and induce remission. The specific treatment regimen may vary depending on the severity of the disease, organs affected, and individual patient factors [132].

The generalized and severe form of the disease (alveolar hemorrhage, glomerulonephritis, heart and CNS involvement, mononeuritis, hearing loss, and leukocytoclastic vasculitis of the skin) requires immediate initiation of induction treatment with high doses of corticosteroids and cyclophosphamide [132]. In large randomized trials, the administration of rituximab, a monoclonal antibody targeting B cells, appeared to have efficacy similar to that of cyclophosphamide [133,134]. Cyclophosphamide may be replaced with rituximab, especially in cases where cyclophosphamide is not suitable due to high risk of toxicity or in relapsing disease [132]. Plasma exchange may be used in severe cases, particularly in patients with rapidly progressive glomerulonephritis or severe renal involvement [16,132]. It helps remove circulating antibodies and inflammatory mediators from the bloodstream. Avacopan, an oral inhibitor of the complement factor C5 receptor, appears to be most beneficial for patients with severe renal disease or alveolar hemorrhage, in addition to cyclophosphamide or rituximab as well as those at higher risk of developing glucocorticosteroid-induced comorbidities [135,136]. Although avacopan is expected to exhibit significant efficacy against renal lesions in MPA/GPA, there is still inadequate evidence to confirm its efficacy against extrarenal lesions [137]. Avacopan was initially not included in the Avacopan for the Treatment of ANCA-Associated Vasculitis (ADVOCATE) trial for patients who required invasive pulmonary ventilation support due to DAH [135]. However, a recent retrospective multicenter case series investigated avacopan use in AAV patients with hypoxic DAH who required oxygen support or mechanical ventilation [138]. After initiating avacopan, DAH resolved in all patients, including those with previously refractory hemorrhaging. Moreover, some patients successfully discontinued corticosteroid use after one month of treatment. With the limitations inherent to retrospective data, future prospective studies are required to evaluate the specific role and optimal timing of avacopan administration in patients with AAV and DAH.

Once remission is achieved, a maintenance therapy regimen is typically initiated to prevent relapse and reduce corticosteroid dosage. This often involves tapering corticosteroids and using less-toxic immunosuppressive agents such as azathioprine, methotrexate, mycophenolate mofetil (MMF), or rituximab [132]. Rituximab has shown superiority over azathioprine in maintaining remission for GPA and MPA following induction therapy with either cyclophosphamide or rituximab when used in conjunction with low-dose glucocorticosteroids [139].

In cases of non-severe disease requiring the induction of remission, there has been a growing utilization of rituximab, with accumulating evidence suggesting its superior efficacy compared to traditional immunosuppressants. However, when rituximab or cyclophosphamide cannot be utilized, methotrexate and MMF may serve as alternative options. Studies indicate that both methotrexate and MMF demonstrate comparable efficacy to cyclophosphamide in achieving remission rates at six months [132]. Nevertheless, they are associated with a higher risk of relapse during follow-up, particularly among patients positive for PR3-ANCA [140].

Antifibrotic treatment may be considered a therapeutic option for patients with ANCA-associated interstitial lung disease [141]. Although no published studies have specifically evaluated the role of antifibrotic agents in AAV, the French Vasculitis Study Group is currently conducting an open-label study to assess the safety and effectiveness of pirfenidone in patients with anti-MPO positivity and pulmonary fibrosis with or without AAV (NCT03385668). While pending for additional evidence, combining immunosuppressive and antifibrotic medications could be a reasonable treatment choice for patients with both fibrosis and AAV, particularly for those with a fibrotic NSIP pattern [142]. However, monotherapy with an antifibrotic agent, e.g., nintedanib or pirfenidone, without immunosuppressive therapy should be evaluated in individuals with AAV without systemic manifestations and a UIP pattern manifesting progressive fibrotic disease [143]. Patients with AAV-ILD have been observed to exhibit less-active vasculitis, yet they bear a higher risk of mortality due to UIP, which progresses independently of vasculitis, and conventional treatments may adversely affect the outcome of UIP [144].

Managing complications such as hypertension, infection, and thrombosis is very important. Prophylactic antibiotics and medications to mitigate osteoporosis risk should be administered to counter the potential side effects of immunosuppressive therapy. Vigilant monitoring of renal function and other organ systems is a vital supportive intervention [132].

If disease relapse occurs, modifications to the treatment plan may be warranted, such as intensifying immunosuppressive therapy or restarting the induction regimen.

The treatment approach for EGPA involves high doses of corticosteroids to rapidly reduce inflammation and alleviate symptoms [20]. Alongside corticosteroids, immunosuppressive medications, such as cyclophosphamide or rituximab, may be employed to help control autoimmune reactions in severe disease [20]. For remission maintenance of non-severe disease or for induction in resistant cases of non-severe EGPA, mepolizumab, which targets IL-5, may be considered [20]. Benralizumab, a fully humanized monoclonal antibody that binds with high affinity to the alpha chain of the IL-5 receptor, and reslizumab, a monoclonal IL-5 antibody, have also shown promising results in remission maintenance in EGPA [145,146,147].

Systemic glucocorticoids, along with supplementary immunosuppressive treatments such as plasma exchange therapy, constitute the primary treatment approach for DAH associated with anti-GBM disease [148,149].

## 6. Perspectives in the Management of Pulmonary Manifestations of AAV

As our understanding of the pathogenesis of AAV and anti-GBM disease has improved, significant advancements have been made in treatment approaches, which have substantially improved remission rates. Future perspectives aiming at improving patient outcomes and quality of life might include [102,132,148]:

- Early diagnosis: Advances in diagnostic methods and prognostic biomarkers may enable earlier detection of vasculitis and its flare-ups, thereby facilitating the timely initiation of treatment and prevention of organ damage.
- Targeted therapies: Identification of the pathways involved in the pathogenesis of AAV and anti-GBM disease may lead to the development of targeted therapies that can selectively inhibit disease-causing mechanisms while leaving normal immune function unaffected. Recently tested medications, such as avacopan, targeting the complement cascade have demonstrated efficacy in reducing overall toxicity and minimizing the need for steroid therapy [135].
- Relapse prevention: Prolonged maintenance therapy may improve long-term outcomes and reduce the burden of disease on patients by lowering the risk of relapse.
- Personalized medicine: The development of personalised treatment plans based on the disease phenotype, genomics, immunological and biomarker profile of each patient may be the result of advances in our understanding of the pathogenesis of AAV-and anti-GBM-disease.

## 7. Conclusions

In individuals predisposed to AAV, various factors, such as infections, drugs, or environmental triggers, can activate innate immunity and cause inflammation [6,102]. This activation leads to the expression of ANCA antigens, such as PR3 and MPO, in neutrophils via pro-inflammatory cytokines and factors. Neutrophils then release inflammatory cytokines and ROS, which can cause damage to small blood vessels [6]. Additionally, the formation of NETs contributes to vascular injury and boosts ANCA production, establishing a harmful feedback loop. An abnormal T cell response characterized by an overabundance of Th1 and Th17 cells, along with inadequate production of regulatory T cells (Tregs), disrupts immune tolerance and triggers the production of autoantibodies against ANCA antigens [6]. These phenomena lead to increased expression of C5a and decreased levels of complement regulators [6].

The pathogenesis of anti-GBM disease involves complex autoimmune mechanisms. Normally, GBM does not provoke an immune response; however, alterations in its hexameric structure can expose cryptic epitopes, triggering an autoimmune response. These epitopes, which can be linear or conformational, are targeted by specific antibodies, leading to inflammation, complement activation, and tissue damage in the kidneys and lungs [118,148]. T cells also play a significant role in disease development, influencing B cell function and antibody production. Environmental factors and genetic predispositions, including certain HLA alleles, can contribute to susceptibility and severity [118,148].

Despite the complexity of their pathogenesis, recent advancements in treatment approaches have significantly improved remission rates, with emerging therapies showing promise for reducing overall toxicity and steroid dependence. Overall, continued research efforts and collaboration among clinicians, researchers, and patients are essential for furthering our understanding of pulmonary manifestations in vasculitis and developing innovative therapies to improve patient care in the future.
